# Supplementary material for: Optimization of human papillomavirus-based pseudovirus techniques for efficient gene transfer
Source: Sci Rep. 2020 Sep 23;10:15517. doi: 10.1038/s41598-020-72027-1 (PMC7511366; doi:10.1038/s41598-020-72027-1)
Supplement: Supplementary file 1 — Supplementary Information. [file 41598_2020_72027_MOESM1_ESM.docx]

**Optimization of human papillomavirus-based pseudovirus techniques for efficient gene transfer**

Timra D. Gilson^1+^, Ryan T. Gibson^2+^ and Elliot J. Androphy^1,2*^

**Supplementary Figure S1.** Serum type effects infection rate. N/TERT cells were grown to confluence, stripped with NH_4_OH buffer, and remaining ECM was incubated with mCherry PsV overnight in media lacking any serum (base) or supplemented with FBS, charcoal stripped FBS, or Horse Serum. N/TERT cells were added next day in fresh F-media and percent red cells was calculated 48 hours post infection. All experiments were performed at least 3 independent times and values shown as mean + SEM.


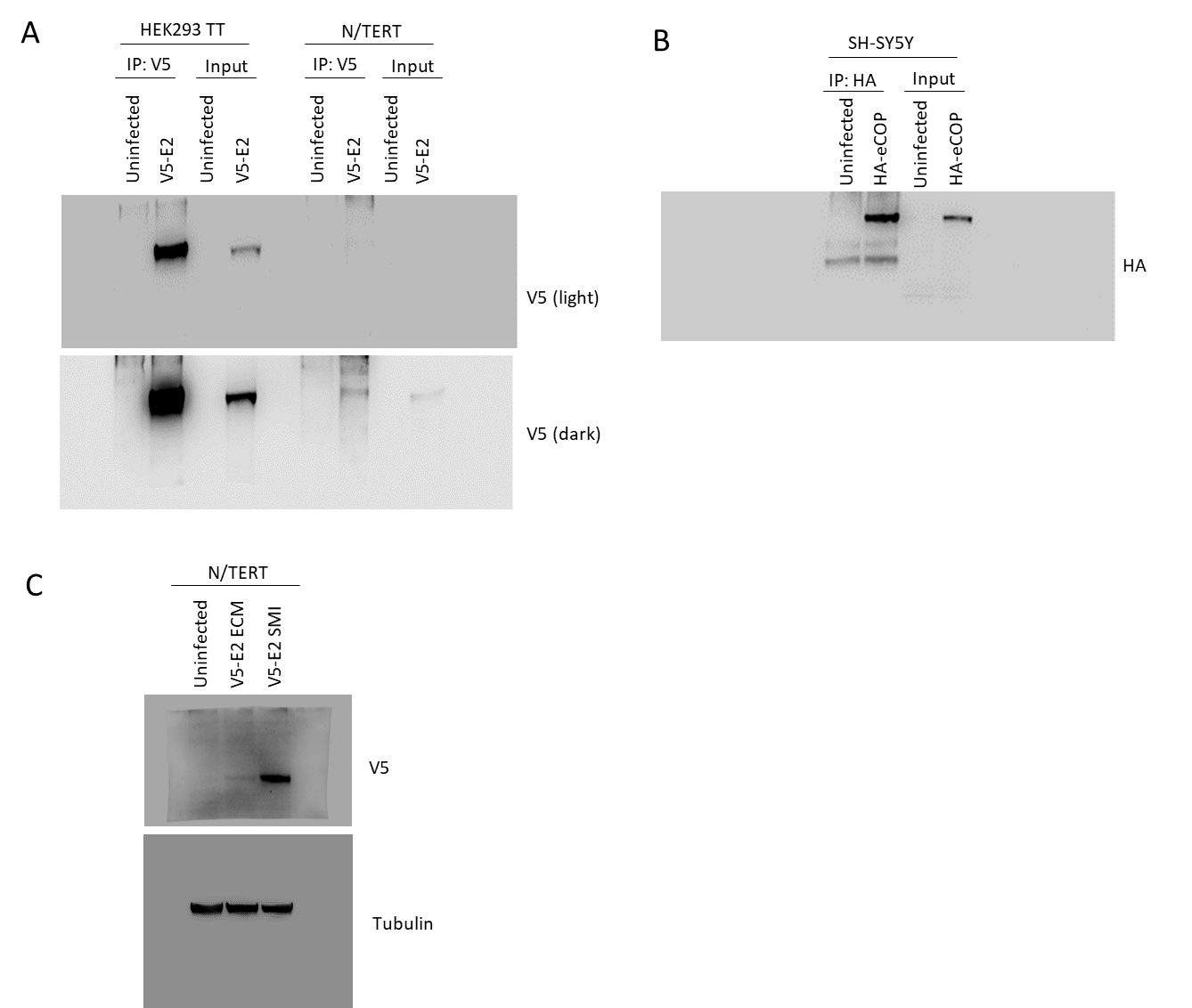


**Supplementary Figure S2.** Full length blots for Figure 7. **(A)** Full length blot for Fig. 7A on left (lighter exposure), and Fig. 7B on right (darker exposure). HEK293 TT or N/TERT cells were infected with HPV-31 V5-E2 PsV for two days, lysed and immunoprecipitated with rabbit anti-V5 antibody, then immunoblotted with mouse anti-V5 antibody. **(B)** Full length blot for Fig 7C. SH-SY5Y cells were infected with HA-εCOP PsV for two days, lysed, and immunoprecipitated with mouse 12CA5A1 anti-HA antibody and immunoblotted with mouse HA-7 anti-HA. **(C)** Full length blot for Fig 7D. N/TERT cells were infected with V5-E2 PsV either by SMI or ECM methods. Two days following infection, cells were harvested and immunoblotted with V5 and Tubulin antibodies.
